# Fighting Mobile Phone Addiction with Forgiveness Following Interpersonal Transgressions: A Psychological Compensation Perspective

**DOI:** 10.3390/bs15091209

**Published:** 2025-09-05

**Authors:** Gang Du, Xiaogang Wang

**Affiliations:** 1 School of Education and Psychology, Southwest Minzu University, Chengdu 610225, China; 2 Key Research Institute of Humanities and Social Sciences of State Ethnic Affairs Commission, Southwest Minzu University, Chengdu 610225, China

**Keywords:** interpersonal transgressions, forgiveness, mobile phone addiction, social bonding, psychological compensation

## Abstract

Current research on addressing mobile phone addiction primarily focuses on mitigating the influence of psychopathological factors. In the present work, we conducted three studies to investigate a novel hypothesis that forgiveness, as a form of psychological compensation, may offer a previously underappreciated protective effect against mobile phone addiction in the aftermath of interpersonal transgressions. Study 1 (*N* = 391), a cross-sectional survey, established an association between negative life events and mobile phone addiction. Utilizing a recall methodology, Study 2 (*N* = 256) demonstrated that higher levels of forgiveness mitigated the adverse effect of interpersonal transgressions on mobile phone addiction. Moreover, forgiveness was particularly effective in reducing mobile phone addiction in cases involving mild—as opposed to severe—transgressions. Subsequently, in Study 3 (*N* = 175), we employed a scenario-based approach to investigate the mechanisms through which forgiveness mitigates mobile phone addiction following interpersonal transgressions. The results indicated that social bonding—specifically, reduced social distance and increased positive affect toward transgressors—mediated the relationship between forgiveness and mobile phone addiction. In future research and practice, forgiveness-based interventions may be leveraged to reduce mobile phone addiction.

## 1. Introduction

With the advancement of information technology, the utilization of mobile phones is becoming increasingly prevalent. Mobile phones, as a double-edged sword, empower individuals to instantaneously share information, access news, and engage in entertainment, thereby enriching quality of life and well-being for many people. A growing body of research has documented the developmentally beneficial aspects of digital tools, particularly in educational supports and neurodiversity contexts (Andreou & Argatzopoulou, 2024; Christou et al., 2023). However, despite these positive utilities, excessive and compensatory usage can lead to various adverse consequences for both healthy development and mental health, such as the emergence of mobile phone addiction (Salehan & Negahban, 2013). Mobile phone addiction, alternatively termed mobile phone dependence or problematic mobile phone use, occurs when individuals lose control over their behavior due to excessive mobile phone usage, leading to a state of obsession (Sahu et al., 2019). Although mobile phone addiction has not been formally classified as an addiction in the fifth edition of the Diagnostic and Statistical Manual of Mental Disorders (DSM-5), it does exhibit characteristics similar to those of other addictive behaviors, such as gambling (Ting & Chen, 2020). It is linked to a range of psychological, social, academic, and occupational issues, including depression, low self-esteem, decreased psychological well-being, social anxiety, attention-deficit/hyperactivity disorder, and loneliness, spanning from adolescence to adulthood (Busch & McCarthy, 2021; Olson et al., 2022; Seo et al., 2016). The mitigation of mobile phone addiction has become a prominent subject of discourse across diverse disciplines. The majority of research progress in addressing mobile phone addiction has been centered around diminishing the impact of psychopathology factors, such as depression, anxiety, chronic stress, low self-esteem (Elhai et al., 2017), and insecure attachment (Y. Zhang et al., 2022). With the growing prominence of positive psychology, individual positive psychological attributes, such as forgiveness, are slowly gaining recognition and undergoing scientific scrutiny. Numerous studies indicate that forgiveness can alleviate or neutralize individuals’ psychological and behavioral responses following offenses or transgressions (Lee & Enright, 2019; Toussaint et al., 2015; Worthington & Wade, 2020). Hence, we were particularly interested in whether forgiveness alleviates mobile phone addiction after interpersonal transgressions.

The causes of mobile phone addiction are multifaceted, with a notable factor being real-life frustration, such as encountering interpersonal transgressions. Existing research indicates that individuals often undergo a cascade of adverse physiological and psychological reactions following interpersonal transgressions, including increases in heart rate, blood pressure, emotional tension, and obsessive thoughts (Berry et al., 2005; Ellett et al., 2023). When the motivation to go online is rooted in an unmet real-life need and internet use helps alleviate the actual problem, individuals may develop a strong inclination to spend more time online, potentially resulting in problematic outcomes (Kardefelt-Winther, 2014). Moreover, mobile phones are significantly easier to access than they were in the past. In the aftermath of interpersonal harm, many retreat into their phones, a fertile ground for addiction to take root. Arslan (2017) empirically reported that psychological maltreatment (a harmful experience) is associated with an increased risk of internet addiction in young adults. Based on these findings, we propose that when individuals experience interpersonal offenses or harm, they are more likely to develop mobile phone addiction (H1). If interpersonal transgressions are likely to increase the propensity for mobile phone addiction, how can we mitigate mobile phone addiction following interpersonal transgressions?

### 1.1. What Compensatory Role Does Forgiveness Fulfill?

As a personal strength and valuable resource, forgiveness has received increasing attention. Currently, there is no consensus in the academic community regarding the definition of forgiveness (Berry et al., 2005; Ho & Fung, 2011; Worthington & Wade, 2020). Various scholars, guided by their academic inclinations and backgrounds, frequently ascribe particular interpretations to the concept of forgiveness that resonates with their scholarly pursuits. Thus far, the academic understanding of forgiveness has predominantly originated from the standpoint of the prosocial transformation taking place within the offender. Of course, forgiveness also includes self-forgiveness and situational forgiveness (Thompson et al., 2005). Forgiveness is a transformative process that entails letting go of negativity toward the transgressor and even potentially cultivating positive feelings and benevolence toward them (Enright & Fitzgibbons, 2015; Forster et al., 2020; McCullough et al., 1998; Worthington & Wade, 2020). Forgiveness is often considered a desirable outcome following conflict (Cowden, 2024), as it contributes to enhanced psychological well-being for both victims and transgressors (Riek & Mania, 2012), as well as improved physiological well-being (Rasmussen et al., 2019) and strengthened relational bonds (Bono et al., 2008). While not always beneficial, such as when it impedes behavioral change (McNulty, 2011), forgiveness is generally viewed as a highly constructive response to victimization (Schumann & Walton, 2022).

According to the developmental compensation hypothesis (W. B. Gao & Chen, 2006), individuals who encounter adverse life circumstances—such as interpersonal difficulties—undergo a process of psychological compensation as part of their normative developmental trajectory. When individuals possess adequate resources to actively address these impediments and fulfill their developmental needs, constructive compensation occurs. Conversely, when psychological resources are insufficient to overcome such obstacles, pathological compensation may arise, manifesting in behaviors such as excessive mobile phone use as a means of compensating for unmet needs. Within this framework, forgiveness can be understood as a form of psychological compensation. Compensation occurs when individuals address a mismatch between their current abilities and environmental demands by devoting greater time and effort, utilizing latent skills, or acquiring new knowledge (Bäckman & Dixon, 1992). Whether facing intentional or unintentional, mild or severe offenses from others, individuals often experience both intrapersonal and interpersonal imbalance, resulting in physical and psychological discomfort (Schumann & Walton, 2022). Fortunately, forgiveness can alleviate such distress. Facilitating forgiveness has been shown to produce beneficial effects in interpersonal contexts, including the regulation of psychological discomfort (Brady et al., 2023), the restoration of social relationships (McCauley et al., 2022), reconnection with the real world, and enhanced social adaptation (Fourie et al., 2020; Hill et al., 2013). Research further indicates that forgiveness may help individuals cope with intense emotional and cognitive distress, such as that associated with substance misuse and suicidality (Webb et al., 2015). Similarly, Arslan (2017) found that forgiveness mediates the relationship between psychological abuse and internet addiction. Based on this evidence, we hypothesize that forgiveness compensates for interpersonal transgressions, thereby reducing mobile phone addiction (H2).

### 1.2. The Mediating Effect of Social Bonding

Why does forgiveness help mitigate mobile phone addiction in the aftermath of interpersonal transgressions? In other words, what compensatory role does forgiveness play? Forgiveness involves reconfiguring or neutralizing negative responses to offenses, which entails letting go of resentment and maintaining a willingness to engage with the transgressor (Forster et al., 2020; McCullough et al., 1998). We propose that forgiveness may reduce mobile phone addiction by reinforcing social bonding. Social bonds refer to the connections and relationships that tie individuals to one another and to broader social structures (Hirschi, 1969). The establishment and maintenance of close social connections are fundamental to human health, well-being, and functioning (Van Monsjou et al., 2015). In fact, social bonding has been likened to a basic human need, comparable to the need for food and water (Baumeister & Leary, 1995). Forgiveness can strengthen this sense of connection following a transgression.

Although much existing research has focused on the intrapersonal benefits of forgiveness—such as enhanced subjective well-being, life satisfaction, and emotional regulation (F. Gao et al., 2022; Riek & Mania, 2012)—less attention has been paid to individuals’ psychological and behavioral responses toward offenders. In today’s society, where interpersonal interactions are increasingly frequent, forgiveness is not merely a personal choice but also a social responsibility that contributes to interpersonal, intergroup, and societal harmony. While often experienced as an individual process, forgiveness is inherently embedded in social contexts (Hook et al., 2008). Therefore, it is important to explore the diverse psychological outcomes of forgiveness within interpersonal interactions, particularly in terms of feelings and behaviors directed toward others. Empirical evidence suggests that forgiveness can amplify positive emotions, improve attitudes toward transgressors, and reduce negative affect (Schumann & Walton, 2022). Over time, it can restore positive relations, promote reconciliation, and facilitate the reintegration of offenders into reciprocal social exchanges, thereby sustaining cooperation (Worthington & Wade, 2020).

Furthermore, strengthened social bonding encourages individuals to engage more actively in real-world social activities, shifting their focus away from virtual environments and reducing time spent on mobile devices. As social beings, humans rely on connections with others for support, resource acquisition, and stress relief—especially during collective crises or disruptions (Tunçgenç et al., 2023). Social networks play a critical role in facilitating adaptive responses and psychological adjustment to new challenges (Houston & Buzzanell, 2018). Several studies directly indicate that social bonding inhibits mobile phone addiction (Al-Kandari & Al-Sejari, 2021; Wan et al., 2022). Thus, we conclude that forgiveness enhances social bonding, which in turn reduces mobile phone addiction. This mechanism will be examined in the present study (H3).

### 1.3. The Present Research

Mobile phone addiction is increasingly recognized as a global public health issue. Grounded in the developmental compensation hypothesis, this study employs a multi-method approach to systematically test hypotheses and provide valuable insights for addressing mobile phone addiction in the contemporary context. Study 1 utilizes a cross-sectional questionnaire survey to examine the relationship between interpersonal transgressions and mobile phone addiction. Study 2 adopts a recall paradigm to investigate whether forgiveness mitigates the impact of interpersonal transgressions on mobile phone addiction—and if so, explores the underlying mechanisms. Study 3 further employs an imagination paradigm to elucidate the mediating role of social bonding in the relationship between forgiveness and mobile phone addiction within contexts of interpersonal transgressions. While prior research has touched upon this topic to some extent, it has largely been theoretical or reliant solely on correlational survey data, without in-depth empirical exploration of the underlying mechanisms. The research protocol was conducted in strict accordance with the ethical principles of the Declaration of Helsinki and received formal approval from the Institutional Review Board of the School of Education and Psychology at Southwest Minzu University.

## 2. Study 1

### 2.1. Participants

Drawing on Monte Carlo simulations, Schönbrodt and Perugini (2013) recommended that a sample size of approximately 250 is advisable for achieving robust correlation estimates. We recruited 430 university students from Sichuan Province, China. After screening, 39 participants did not meet the inclusion criteria and were consequently excluded. This process resulted in a final sample of 391 participants (179 men, 212 women; *M*_age_ = 20.03 years, *SD*_age_ = 1.52). Permission was obtained from the respective universities, and informed consent was acquired from all participants. We remunerated each participant with a small gift for their participation.

### 2.2. Measures

Interpersonal Transgressions The Adolescent Life Events Scale (ASE) developed by Liu et al. (1997) was used in this study. This self-report questionnaire comprises 27 items representing negative life events that may trigger psychophysiological responses in adolescents. These events are categorized into six dimensions: interpersonal relationships (utilized in our analysis), academic pressure, punishment, loss, health and adaptation issues, and others. Participants assess five events by first confirming each event’s occurrence within the past twelve months. If the event had not occurred, they assigned it a score of 0. If the event did occur, they rated their psychological response on a 5-point scale (1 = none to 5 = very severe) based on their feelings at the time of the event. In this study, the Cronbach’s α coefficient for this scale was 0.75.

Mobile Phone Addiction To measure mobile phone addiction, we employed a validated 16-item scale (Xiong et al., 2012) assessing four components: withdrawal symptoms, salience, social comfort, and mood changes. Sample items included concerns about device functionality (e.g., “I get anxious when my phone is about to die”). Responses were recorded on a 5-point agreement scale, with elevated composite scores signaling stronger addiction. The scale showed high reliability (α = 0.92) in our sample.

### 2.3. Results

Data analysis was performed using SPSS (version 26.0; IBM Corp., Armonk, NY, USA). The results, including means, standard deviations, and correlation coefficients, are presented. Correlation analysis revealed that the dimensions of interpersonal relationships (*M* = 1.86, *SD* = 0.71) were significantly correlated with mobile phone addiction (*M* = 2.48, *SD* = 0.62) (*r* = 0.29, *p* < 0.001). Furthermore, the regression analysis revealed that after controlling for age and sex, the dimensions of interpersonal relationships predicted mobile phone addiction (*B* = 0.27, *t* = 5.72, *p* < 0.001, 95% confidence interval [CI] = [0.19, 0.35]), supporting H1.

An initial questionnaire study (Study 1) established that negative relationships are a significant predictor of mobile phone addiction. Following this discovery, we employed a recall methodology in a subsequent study to investigate the potential buffering effect of forgiveness on the relationship between interpersonal transgressions and mobile phone addiction.

## 3. Study 2

### 3.1. Participants

The calculations performed using G*Power 3.1 (Faul et al., 2007) indicated that for the multifactor analysis of variance applied in this study, a minimum total sample size of 128 was needed to achieve 80% statistical power at a significance level of α = 0.05, assuming a medium effect size (*f* = 0.25). We recruited a total of 279 participants. However, 23 participants did not meet the inclusion criteria, resulting in a final sample of 256 participants. The participants had an average age of 32.10 years (*SD* = 10.88), and 140 of them were female (54.7%). Informed consent was obtained from the participants, and each participant received compensation of CNY 2.00 for their involvement.

### 3.2. Measures

Forgiveness Manipulation Given that transgressions can range from mild to severe and that the severity of harm is significantly negatively related to forgiveness (Fehr et al., 2010; Riek & Mania, 2012; Wang et al., 2024), a 2 (type of transgression: severe transgressions vs. mild transgressions) × 2 (degree of forgiveness: forgiveness vs. unforgiveness) between-subjects design was employed, with mobile phone addiction as the dependent variable. In the severe transgression condition, participants were instructed as follows: “Please recall a recent incident from the past month that deeply offended or harmed you and provide a detailed description of the event”. In the mild transgression condition, participants were instructed as follows: “Please recall a recent incident from the past month that resulted in mild offense or harm to you and provide a detailed description of the event”. In the forgiveness condition, participants were instructed to affirm the following statement: “I have actively forgiven the person who offended or harmed me and try to get along harmoniously with him or her”. In the unforgiveness condition, participants were instructed to affirm the following statement: “I will not forgive the person who offended or harmed me and hope that he or she pays for it” (McCullough et al., 1998).

Mobile Phone Addiction Eight scale items demonstrating validity for measuring episodic smartphone addiction were extracted from Study 1. These included statements such as “I favor mobile messaging over physical social interaction” and “I experience distress during extended phone abstinence periods.” The items were scored on a 5-point scale (1 = strongly disagree, 5 = strongly agree). In this study, the Cronbach’s α coefficient for this scale was 0.91.

Manipulation Check Two items measured the manipulation effects of the type of transgression: “Please rate the severity of the event you wrote about” and “Please rate the impact of the event you wrote about”. Both items were scored on a 5-point scale (1 = strongly disagree, 5 = strongly agree). Two items measured the manipulation effects of the degree of forgiveness: “Please rate the extent to which you forgive the person who offended or harmed you in the event you wrote about” and “You want to see the person who offended or harmed you in the event you wrote about suffering and in pain”. Both items were also scored on a 5-point scale (1 = strongly disagree, 5 = strongly agree).

Procedure Our study was conducted online using Credamo (https://www.credamo.com). After providing online informed consent and demographic information, the participants were randomly assigned to one of four manipulation conditions (severe-forgiveness, *n* = 63; severe-unforgiveness, *n* = 73; mild-forgiveness, *n* = 64; mild-unforgiveness, *n* = 56). Participants were instructed to dedicate a few minutes to thoughtfully recollect and document the event with precision. Subsequently, they were tasked with reading instructions pertaining to either forgiveness or unforgiveness. Participants subsequently completed the mobile phone addiction questionnaire and the manipulation check questionnaire.

### 3.3. Results

First, a manipulation check was conducted. The results indicated that participants in the severe transgression condition rated the severity of the event (*t* = 5.85, *p* < 0.001; Cohen’s *d* = 0.73) and the level of impact (*t* = 5.41, *p* < 0.001; Cohen’s *d* = 0.68) significantly greater than did those in the mild transgression condition. Participants in the forgiveness condition showed a significantly greater level of forgiveness toward the offenders than did those in the unforgiveness condition (*t* = 7.62, *p* < 0.001, Cohen’s *d* = 0.95), and they also reported a significantly lower level of desire for punishment toward the offenders (*t* = −6.08, *p* < 0.001, Cohen’s *d* = −0.76) than did those in the unforgiveness condition.

**Figure 1:**
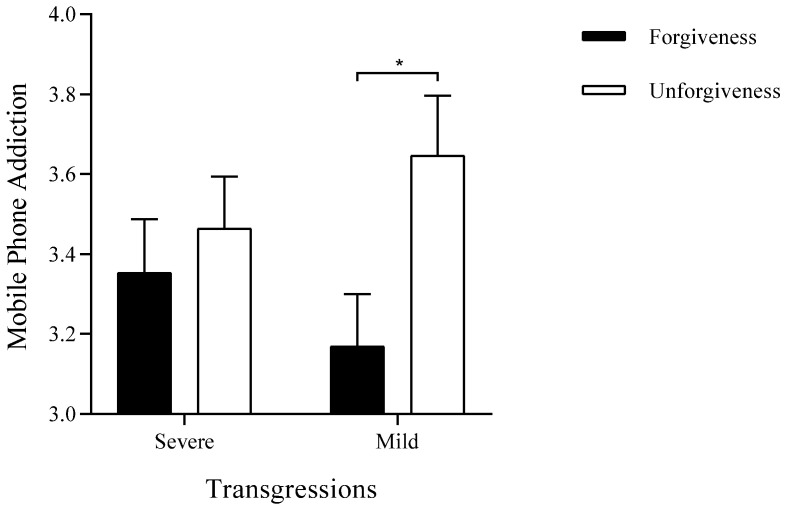
Buffering effect of forgiveness on mobile phone addiction under interpersonal transgressions. Note: * *p* < 0.05.

Next, to test the buffering effect of forgiveness on mobile phone addiction following interpersonal transgressions, a one-way ANOVA was conducted with mobile phone addiction as the dependent variable. The results (see Figure 1) indicated that, while controlling for sex, age, education level, and personal annual income, the main effect of the type of transgression was not significant (*F*(1, 248) = 0.01, *p* = 0.98, η^2^_p_ = 0.00). However, the main effect of the degree of forgiveness was significant (*F*(1, 248) = 11.85, *p* < 0.01, η^2^_p_ = 0.05). Mobile phone addiction in the forgiveness condition (*M* = 3.20, *SD* = 0.99) was significantly lower than that in the unforgiveness condition (*M* = 3.60, *SD* = 0.89). The interaction between the type of transgression and the degree of forgiveness was significant (*F*(1, 248) = 4.16, *p* < 0.05, η^2^_p_ = 0.02). Further simple effects analysis showed that under the severe transgression condition, there was no significant difference in mobile phone addiction between the forgiveness condition (*M* = 3.32, *SD* = 1.05) and the unforgiveness condition (*M* = 3.49, *SD* = 0.87, *t*(134) = −1.02, *p* = 0.31, Cohen’s *d* = 0.18). However, under the mild transgression condition, mobile phone addiction in the forgiveness condition (*M* = 3.07, *SD* = 0.91) was significantly lower than that in the unforgiveness condition (*M* = 3.70, *SD* = 0.91, *t*(118) = −3.74, *p* < 0.01, Cohen’s *d* = 0.69). This finding suggested that, compared to severe transgression conditions, forgiveness mitigates mobile phone addiction under mild transgression conditions.

While Study 2 makes valuable contributions building upon Study 1, it still has several limitations, such as the lack of exploration of the underlying mechanisms involved. Study 3 further investigates the potential mechanisms through which forgiveness influences mobile phone addiction, with a focus on the mediating role of social bonding. Another limitation of Study 2 is that the events recalled by individuals are based on their own life experiences. Although this method captures various offensive or harmful events under natural life circumstances, these events have different attributes and elicit varying psychological responses for individuals, which can impact the generalizability of the research findings (Adams & Inesi, 2016). Moreover, individuals’ current feelings and attitudes can influence their evaluation and recall of past events (Schumann & Walton, 2022). Therefore, in subsequent research, we will consider the effects of limiting participants to imagining or experiencing the same offensive or harmful event in a controlled environment to validate the research hypotheses.

## 4. Study 3

### 4.1. Participants

According to calculations derived using G*Power 3.1 (Faul et al., 2007), the multiple linear regression model applied in this study requires a minimum total sample size of 128 to achieve 80% statistical power at a significance level of α = 0.05, assuming a medium effect size (*f* = 0.25). We recruited a total of 175 participants through both offline and online platforms, including Credamo (https://www.credamo.com). However, 25 participants did not meet the inclusion criteria, resulting in a final sample size of 150 participants. The average age of the participants was 25.27 years (*SD* = 12.03), and 77 of them were female (51.3%). Informed consent was obtained from the participants, and each participant received compensation of CNY 2.00 for their involvement.

### 4.2. Measures

Forgiveness Manipulation We manipulated forgiveness using a modified event-reflection task. Participants were presented with the following scenario: “A colleague told me that they had to submit their project proposal before the weekend. I had already completed my proposal. The colleague mentioned that they had not yet figured out what to write and asked whether they could use my proposal as a reference, promising to make their proposal different from mine. I agreed. However, without my knowledge, my colleague submitted my proposal after making some modifications. The supervisor noticed two identical proposals and accused me of plagiarizing the colleague’s work, reprimanding me harshly in their office and threatening to disqualify me from the project. I explained my thought process and the project analysis to the supervisor, while the colleague had no knowledge of the proposal’s content. The supervisor eventually believed that I had written the proposal” (Chen et al., 2017). For the forgiveness condition, participants read a passage that concluded with a forgiveness-inducing statement: “I forgave the colleague and, considering the punishment of disqualification from the project was too severe, I voluntarily suggested to the supervisor to give the colleague a chance to redo their proposal”. In the unforgiveness condition, the materials concluded with an unforgiveness statement: “I still insisted on a severe punishment for the colleague who copied the proposal”.

Social Bonding To ensure the stability of the results, social bonding was measured using two approaches. First, psychological distance from the offender was assessed using the Interpersonal Relationship Scale (Aron et al., 1992), which measures the state-level colleague relationship based on the degree of circle overlap to reflect psychological distance. Responses were collected using a 7-point Likert scale, where elevated scores corresponded to increased levels of perceived intimacy. Second, affective bonding with the offender was measured using affect vocabulary, following the approach of Wood and Kenyon (2018). Affective bonding characteristics were assessed using affect words. Specifically, the study incorporated four emotion terms (representing happiness, sadness, pride, and anxiety) adapted from the well-validated PANAS measure (Watson et al., 1988). Participants were asked to report their affective experiences regarding their relationship with their colleague using items such as “The relationship between me and that colleague makes me feel happy/sad/proud/anxious”. This approach aimed to measure participants’ levels of both positive and negative affective bonding with their colleague on a scale ranging from 1 to 5.

Mobile Phone Addiction Consistent with the methodology employed in Study 2, smartphone dependency was assessed using identical measurement procedures. The instrument demonstrated excellent internal consistency in the current sample (α = 0.92).

Manipulation Check The manipulation check for forgiveness was conducted using the same method as that used in Study 2. The second item was reverse-scored.

Procedure After providing online informed consent and demographic information, participants were allotted up to 5 min to review the scenario. After reading the scenario, the participants were randomly assigned to either the forgiveness (*n* = 74) or unforgiveness (*n* = 76) manipulation group. The participants subsequently completed the social bonding questionnaire, mobile phone addiction questionnaire, and manipulation check questionnaire.

### 4.3. Results

#### 4.3.1. Manipulation Check

The manipulation check for forgiveness revealed that the average scores of the two items in the forgiveness condition (*M* = 4.16, *SD* = 1.68) were significantly greater than those in the unforgiveness condition (*M* = 2.45, *SD* = 1.57, *t*(148) = 6.44, SE = 0.27, *p* < 0.001, Cohen’s *d* = 1.05). These results indicate the effectiveness of our forgiveness manipulation.

#### 4.3.2. Social Bonding and Mobile Phone Addiction Under Different Manipulation Conditions

We examined the differences in social bonding and mobile phone addiction among participants in different manipulation conditions (see Table 1). The results revealed that, compared to those in the unforgiveness condition, participants in the forgiveness condition had a closer social distance (*t*(148) = 2.01, SE = 0.28, *p* < 0.05, Cohen’s *d* = 0.33, 95% CI [0.00, 0.65]); had more positive affect toward the transgressor (*t*(148) = 3.53, SE = 0.16, *p* < 0.001, Cohen’s *d* = 0.58, 95% CI [0.24, 0.91]); and experienced less negative affect toward the transgressor (*t*(148) = −3.00, SE = 0.20, *p* < 0.01, Cohen’s *d* = −0.49, 95% CI [−0.82, −0.16]). Furthermore, in the forgiveness condition, participants exhibited a lower level of mobile phone addiction than did those in the unforgiveness condition (*t*(148) = −2.01, SE = 0.15, *p* < 0.05, Cohen’s *d* = −0.33, 95% CI [−0.65, 0.00]).

#### 4.3.3. Mediation Analysis

**Figure 2:**
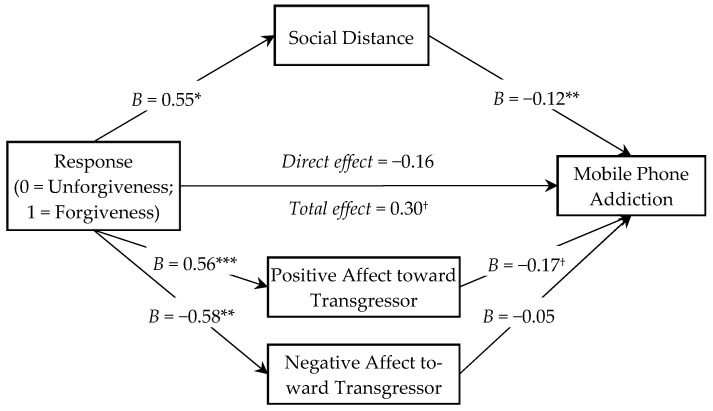
The mediation model. Note: Path coefficients were unstandardized. ^†^ *p* < 0.1, * *p* < 0.05, ** *p* < 0.01, *** *p* < 0.001.

We used the SPSS macro program “Process” (Hayes, 2013) to test the mediating effect of social bonding. We coded the forgiveness group as 1 and the unforgiveness group as 0. In the model, demographic characteristics (sex, age, education level, and personal annual income) were controlled, while forgiveness was specified as the independent variable, mobile phone addiction use as the dependent variable, and social bonding as the mediator. In this process, we selected Model 4 to test the mediating effect. The results indicated that the model was significant (*R*^2^ = 0.16, MSE = 0.79, *F*(6, 143) = 4.51, *p* < 0.001), as shown in Figure 2. Forgiveness significantly predicted social distance (*B* = 0.55, SE = 0.28, *t* = 2.00, *p* < 0.05, 95% CI [0.01, 1.09]), positive affect toward the transgressor (*B* = 0.56, SE = 0.15, *t* = 3.58, *p* < 0.001, 95% CI [0.25, 0.87]), and negative affect toward the transgressor (*B* = −0.58, SE = 0.19, *t* = −3.06, *p* < 0.01, 95% CI [−0.95, −0.20]). Social distance significantly predicted mobile phone addiction (*B* = −0.12, SE = 0.04, *t* = −2.79, *p* < 0.01, 95% CI [−0.21, −0.04]). The prediction of mobile phone addiction by positive affect toward the transgressor marginally reached significance (*B* = −0.17, SE = 0.09, *t* = −1.88, *p* = 0.06, 95% CI [−0.34, 0.00]), and the prediction of mobile phone addiction by negative affect toward the transgressor was not significant (*B* = −0.05, SE = 0.07, *t* = −0.64, *p* = 0.52, 95% CI [−0.20, 0.10]). The direct effect of forgiveness on mobile phone addiction was not significant (*B* = −0.16, SE = 0.15, *t* = −1.04, *p* = 0.30, 95% CI [−0.47, 0.15]), and the total effect on mobile phone addiction was marginally significant (*B* = −0.30, SE = 0.15, *t* = −1.97, *p* = 0.05, 95% CI [−0.59, 0.00]). The analysis revealed that both social distance and positive affect toward the transgressor mediated the association between forgiveness and mobile phone addiction, explaining 45.7% of the total variance. The indirect effect through social distance was −0.07 (95% CI [−0.21, 0.00]), while the mediating effect of positive affect toward the transgressor was −0.10 (95% CI [−0.24, −0.01]).

## 5. Discussion

Our multi-study investigation examined: (1) the association between interpersonal transgressions and mobile phone addiction (H1), (2) the potential buffering role of forgiveness in this relationship (H2), and (3) forgiveness-mediated pathways leading to mobile phone addiction (H3). The findings provide novel insights into the dynamics underlying mobile phone addiction. Study 1, using naturalistic data, supports H1 by revealing that distressing interpersonal events significantly predicted mobile phone addiction. Building on this, Study 2 demonstrates that forgiveness mitigates the risk of mobile phone addiction following such transgressions, thereby supporting H2. Further elucidating this mechanism, Study 3 shows that forgiveness acts as a compensatory factor that promotes social bonding, offering empirical support for H3. Collectively, the evidence dynamically illustrates that mobile phone addiction is particularly heightened in response to interpersonal transgressions (Mao et al., 2023), and aligns with the conceptualization of forgiveness as a prosocial transformation from hostility to friendliness (Forster et al., 2020; McCullough et al., 1998).

Study 1, conducted via questionnaire survey, revealed that individuals experiencing negative life events tend to exhibit higher levels of mobile phone addiction. Within a cognitive-behavioral framework, Davis (2001) conceptualized internet addiction through a diathesis-stress model, positing that maladaptive online behaviors arise from the interaction between preexisting vulnerability and stressful life circumstances. Such negative events are typically characterized by their disruptive nature, often eliciting cognitive dissonance, depressed mood, heightened irritability, and other health-related and psychological issues (Berry et al., 2005; Ellett et al., 2023). According to the compensatory internet use theory (Kardefelt-Winther, 2014), adverse life situations may motivate individuals to engage extensively with online activities as a strategy to alleviate negative emotions. Consequently, people often turn to alternative sources of satisfaction—such as socializing or entertainment on their mobile phones—which are easily accessible and offer an immediate escape from real-life challenges. From a clinical perspective, cognitive behavioral therapy (CBT) can be effective in reducing mobile phone addiction by targeting and modifying irrational or distorted cognitions related to negative events (Burkholder, 2022). When external circumstances are unchangeable, shifting individuals’ perceptions and attitudes toward these events can lead to meaningful improvements in psychological and behavioral health, including reductions in addictive mobile phone use.

Furthermore, social support and psychological resilience can shape how individuals interpret and cope with stressful experiences (Smith et al., 2017). Among positive psychological resources, forgiveness has received extensive theoretical and empirical attention for its role in promoting health and well-being (Worthington & Wade, 2020). In line with H2, our Study 2 demonstrated that higher levels of forgiveness buffer the negative impact of interpersonal transgressions on mobile phone addiction. Previous studies have shown that forgiveness indirectly predicts lower depression through improved emotion regulation (L. Zhang et al., 2020) and mitigates depressive symptoms among emerging adults who experience phubbing (Xie et al., 2020). Building on this foundation, our research further indicates that forgiveness not only reduces negative affect but also shapes subsequent behavioral outcomes—such as decreasing the risk of addictive behaviors or suicidal tendencies (Webb et al., 2015). This focus on the behavioral consequences of forgiveness represents an important advancement beyond prior work. These findings highlight the practical value of implementing targeted forgiveness interventions within counseling, school programs, and digital well-being education to alleviate compulsive phone use stemming from interpersonal conflicts. Supporting this approach, a meta-analysis of randomized controlled trials (RCTs) confirmed that process-based forgiveness interventions—such as the REACH model—are effective in reducing depression, anger, hostility, and stress, while also promoting positive emotions (Akhtar & Barlow, 2018).

Simple effects analysis indicated that under conditions of mild interpersonal transgression, individuals in the forgiveness condition scored significantly lower on mobile phone addiction than those in the unforgiveness condition. However, Study 2 did not observe a significant conciliatory effect of forgiveness on mobile phone addiction in cases of severe transgression—although mean addiction scores in the forgiveness condition were still numerically lower than those in the unforgiveness condition. This lack of a significant effect under severe transgression may be attributable to the greater difficulty of eliciting genuine forgiveness in such contexts (Wang et al., 2024), thereby limiting its effectiveness in mitigating addictive behavior. Previous meta-analyses have consistently demonstrated that forgiveness is negatively associated with both the severity of harm and the motivation for reconciliation (Fehr et al., 2010; Riek & Mania, 2012). Thus, forgiveness may not represent the most effective mechanism for reducing mobile phone addiction among individuals who have experienced severe interpersonal transgressions. Additionally, given that the reliance on self-reported recall of past transgressions and emotions may introduce recall bias, future research would benefit from incorporating objective behavioral metrics—such as tracked screen time and application usage statistics—alongside self-report measures. This integrated approach would offer a more comprehensive and robust assessment of mobile phone usage patterns.

Building on the findings of Study 2, Study 3 further demonstrated that social bonding mediates the relationship between forgiveness and reduced smartphone overuse. From an evolutionary perspective, social connections are fundamental to human reproduction and survival (Baumeister & Leary, 1995), which underscores the broad relevance of these results across practical contexts. This study proposes that forgiveness facilitates social bonding through both cognitive and affective pathways, which in turn mitigates mobile phone addiction. Cognitively, forgiveness reduces perceived social distance between individuals, encouraging victims to reconnect with offenders and thereby repair relational rifts caused by transgressions. Previous research has established that closer interpersonal relationships promote forgiveness (Karremans et al., 2011); our findings extend this work by suggesting that forgiveness itself enhances perceived closeness, indicating a mutually reinforcing cycle between relational intimacy and forgiveness capacity. Forgiveness can thus be viewed as an active process of cultivating positive responses toward oneself and others (Arslan, 2017). Affectively, our data show that forgiveness enhances positive feelings toward the transgressor, which consequently contributes to a reduction in mobile phone addiction. Prior studies indicate that forgiveness offers greater emotional benefits than retaliation—such as reduced anger and fewer negative emotions (Chen et al., 2017)—and promotes positive affect both toward others and oneself (Schumann & Walton, 2022). Thus, forgiveness entails not only personal emotional gains but also elements of altruism. Notably, this study did not find a mediating role of negative affect toward transgressors, which may be due to the stronger emphasis on cognitive (rather than affective) processes in scenario-based methodologies (Fehr et al., 2010). Our experimental scenario study focused on generalized responses. It is likely, however, that individual differences—such as trait forgiveness, as measured by instruments like the Heartland Forgiveness Scale—as well as cultural differences serve as important boundary conditions that moderate the observed effects. Future research should examine these factors in greater detail. Overall, these findings align with the theoretical view that although the efficacy of forgiveness may be limited in cases of severe harm, it serves as a critical compensatory mechanism that strengthens social bonds and thereby reduces mobile phone addiction in the aftermath of interpersonal transgressions.

Previous research has predominantly focused on symptom reduction and psychopathological factors contributing to mobile phone addiction (Elhai et al., 2017), often overlooking the role of positive psychological resources. Through both theoretical and empirical investigation, this study identifies forgiveness as a potential protective factor that can help mitigate mobile phone addiction. Although the psychological and physical benefits of forgiveness have been widely documented, its interpersonal functions and underlying mechanisms remain underexplored. Our findings indicate that forgiveness significantly alleviates interpersonal tensions and enhances relationship quality. By engaging in forgiveness, individuals can repair and strengthen social bonds (Forster et al., 2020; McCullough et al., 1998), thereby compensating for the relational disruptions caused by interpersonal transgressions. As the saying goes, “No man is an island”—forgiveness helps restore damaged relationships, enabling affected individuals to re-engage in mutually beneficial real-world interactions and reducing their reliance on mobile phones for emotional escape. That said, forgiveness should not be viewed as a standalone solution. It is most effective when integrated with other coping strategies, such as setting digital boundaries, building robust support systems, and seeking professional guidance when necessary. By offering a multifaceted support framework, we can better assist those affected by interpersonal harm in healing emotional wounds and reducing their overreliance on the virtual world.

Despite the contributions of this study, several limitations should be acknowledged. First, our research focused exclusively on forgiveness from the victim’s perspective and did not consider the role of the offender. Previous work has highlighted distinctions between forgiveness granted by victims and forgiveness sought by perpetrators (Adams & Inesi, 2016), suggesting that the psychological experiences of each party are not equivalent. Future studies should incorporate both perspectives to provide a more comprehensive understanding of interpersonal forgiveness dynamics. Second, forgiveness is not universally appropriate across all contexts (Boon et al., 2025). As demonstrated in our findings, forgiveness did not significantly reduce mobile phone addiction in cases of severe transgression. Moreover, unforgiveness—including retaliatory responses—may serve functional or even necessary roles in certain situations (Jackson et al., 2019). Thus, future research should investigate the boundary conditions under which forgiveness and unforgiveness are most adaptive. Third, while our results suggest that forgiveness can mitigate mobile phone addiction, the reverse causal pathway remains unexplored. It is plausible that individuals with pronounced phone addiction—who may neglect real-world relationships—exhibit lower levels of forgiveness. Longitudinal designs are needed to clarify the dynamic and potentially bidirectional relationships among these variables. Finally, this study examined social bonding as one mediating mechanism linking forgiveness to reduced mobile phone addiction. However, the etiology of mobile phone addiction is multifactorial, influenced by a range of motivational, cultural, and contextual factors. Further research should investigate additional mediators and moderators—such as individual differences in motivation or cultural background—within the broader framework of forgiveness as a compensatory response to interpersonal transgressions. Furthermore, pursuing alternative explanatory models (e.g., emotion-focused coping and attentional control) would be highly beneficial to future research.

In conclusion, the present study provides empirical support for the role of forgiveness as a psychological compensation that can buffer against mobile phone addiction in the aftermath of interpersonal transgressions. Furthermore, this research highlights the pivotal mediating role of social bonding in explaining how forgiveness reduces compulsive phone use, underscoring the importance of nurturing and restoring interpersonal connections. These findings contribute to a deeper understanding of how positive psychological processes can mitigate maladaptive technology-related behaviors and promote healthier social functioning in the digital age.

## Figures and Tables

**Table 1:** Descriptive statistics and difference tests.

	Response Conditions		*t*	*p*	Cohen’ *d*	95% CI
	Forgiveness *M* (*SD*)	Unforgiveness *M* (*SD*)				
Social distance	3.99 (1.71)	3.43 (1.66)	2.01	<0.05	0.33	[0.00, 0.65]
Positive affect toward Transgressor	2.56 (1.04)	1.99 (0.95)	3.53	<0.001	0.58	[0.24, 0.91]
Negative Affect toward Transgressor	2.98 (1.22)	3.57 (1.18)	−3.00	<0.01	−0.49	[−0.82, −0.16]
Mobile Phone addiction	2.42 (0.90)	2.73 (0.98)	−2.01	<0.05	−0.33	[−0.65, 0.00]

## Data Availability

The data will be available from the corresponding author upon reasonable request.
